# Aptamer-functionalized AuNPs for the high-sensitivity colorimetric detection of melamine in milk samples

**DOI:** 10.1371/journal.pone.0201626

**Published:** 2018-08-02

**Authors:** Xinran Hu, Keke Chang, Shun Wang, Xiaoquan Sun, Jiandong Hu, Min Jiang

**Affiliations:** 1 School of Biological Sciences, The University of Hong Kong, Hong Kong SAR, China; 2 College of Mechanical and Electrical Engineering, Henan Agricultural University, Zhengzhou, China; 3 State Key Laboratory of Wheat and Maize Crop Science, Zhengzhou, China; 4 Flow Measurement Institute, Henan institute of metrology, Zhengzhou, China; 5 College of Life Sciences, Henan Agricultural University, Zhengzhou, China; Institute of Materials Science, GERMANY

## Abstract

Although aptamer-functionalized AuNPs technology exhibits great potential in analytical and biological chemistry, direct analysis of molecules at a low concentration using colorimetric assay remains challenging. The development of intuitive methods has attracted interest for promising detection of melamine in milk samples due to a demand for stable and understandable process. In this study, we propose a rapid and facile colorimetric measurement method of melamine combined aptamer-functionalized AuNPs in contaminated milk samples. To realize the high stability and the lower limit of detection, the aptamer-functionalized surface of AuNPs via a coordinated bond was used in combination with ultra-sonication. The kinetics of this colorimetric assay based on aptamer-functionalized AuNPs was analyzed to illustrate that the higher the concentration of melamine, the faster the aggregation of AuNPs induced. The sensitivity, selectivity, limit of detection and recovery rate were sufficiently validated to understand the measurement principle of melamine using aptamer-functionalized AuNPs. The calibration curve established by the absorption peak ratio (A640 /A520) was linear in the concentration range of 0~1μM of melamine in aqueous solutions with the correlation coefficient (R^2^) of 0.986 and the limit of detection (LOD) of 22 nM, whereas, the correlation coefficient (R^2^) of 0.998 and the LOD of 14.9 nM were achieved at the concentration of melamine below 0.5 μM in milk samples. The optimized performance of this colorimetric assay of melamine using aptamer-functionalized AuNPs in milk samples was obtained with 100 μL of 13 nm AuNPs solution, 40 μL of 1 μM (100 dilutions) aptamers and the pre-reaction time of 30 min. This simple colorimetric measurement of melamine using aptamer-functionalized AuNPs provides a promising target for various applications of the sample source with complex sample matrices.

## Introduction

Melamine is an organic compound that is commonly found in the form of nitrogen-rich white crystals, and is synthesized from urea decomposing into cyanic acid with an intermediate step. Sometimes, they have been illegally added into food, animal feed and even milk to increase the apparent protein content [1, 2]. High-dosage melamine will result in severe kidney damage to both humans and animals. When other impurities associated with melanin synthesis appear, particularly cyanuric acid, the toxicity of melamine is further aggravated [3]. In the past two decades, food safety problems caused by melamine contamination have emerged in an endless stream. Therefore, detection of melamine has always been one of the hot issues of research for each country in the world. In practical, some conventional methods have already been employed in laboratory have already been utilized to detect melamine, such as capillary electrophoresis[4], chemiluminescence [5], electrochemical immunosensors [6], high performance liquid chromatography/high performance liquid chromatography-mass spectrometry (HPLC/HPLC-MS)[7–9] and antibody-based immunological method [10]. Moreover, these conventional methods require expensive equipment and trained operators. The corresponding sample preparations are time-consuming and tedious. Therefore, it is highly desirable to develop a new rapid detection method to replace these traditional methods. Recently, gold nanoparticles (AuNPs) have been shown to possess many attractive characteristics including unique optical and electrical properties as well as a high molar extinction coefficient. These features allow them to serve as sensitive probes for detecting chemical and biological analytes [11]. AuNPs are also easy to prepare in a liquid by reducing chloroauric acid with a reductant [12, 13]. However, the size and shape of AuNPs are closely related to the detection sensitivity. Actually, the influence produced by the size and shape of AuNPs used in colorimetric assay has been investigated by many researchers [14–16]. For example, the spectral width of AuNPs is decreased with the increasing size of AuNPs [17, 18]. The aqueous-based seed-mediated growth of monodisperse gold octahedra with wide range of sizes (50–150 nm in side length) has been investigated to reduce different amounts of HAuCl_4_ using butenoic acid as reducing agent. These plasmonic substrates exhibit high and uniform SERS signals over extended areas with intensities increasing with the size of Au nanoparticle [19]. The synthesis of nearly monodisperse single crystalline silver nanocubes in a non-polar solvent (1,2-dichlorobenzene) using oleylamine as the reducing and capping agent has been investigated[20]. Moreover, AuNPs have the potential to be applied on-site monitoring of environmental pollution due to the intrinsic absorption spectroscopy, which is sensitive to the change of extinction coefficient caused by the aggregation of AuNPs [21]. These approaches have demonstrated the excellent analytical performance of AuNPs with a high sensitivity and excellent selectivity as driven by the interactions of the analyte-AuNPs surroundings, such as electrostatic, hydrogen bonds, and so on [22]. One of the most common applications of biological analytes is to encapsulate the AuNPs with a functional group or molecules. The encapsulated agent should be able to bind specifically with the analytes and dissociate from the AuNPs, thereby allowing the aggregation of AuNPs induced by salt. This mechanism was associated with localized surface plasmon resonance (LSPR) and has been adopted in detecting ochratoxin A (OTA) [23–26]. A series of colorimetric sensors using the encapsulated AuNPs have been proposed for rapid detection of other hazardous materials [27–35]. A novel colorimetric sensing method for detection of plant hormone abscisic acid (ABA) was validated by our group. Measuring occurrences of the ABA molecules directly and repeatedly, highly specific nucleic acid aptamers enable molecular recognition of ABA to form the ABA-aptamer complexes with G-quadruplex structures which couple a common scaffold to lose the ability that stabilizes AuNPs against NaCl-induced aggregation in ABA recognition [36]. Recently, some papers have reported on the development of colorimetric sensors for detecting melamine [37–44]. Although melamine in milk products has been detected on-site and real-time by using a reliable and highly sensitive colorimetric sensing method significantly involving the aggregation of AuNPs, the colorimetric assay of melamine using AuNPs in our recently published paper is found to be susceptible to complex solution conditions [45]. Therefore, it is imperative to explore a new, efficient and stable method to detect melamine in milk samples. To achieve this goal, the surface of AuNPs needs to be functionalized with melamine aptamers via the coordinated bond, which allows aptamers to specifically bind the melamine to trigger the aggregation of AuNPs in the presence of salt [46]. Being aware of these research areas in functionalized AuNPs, some researchers have already reported on the sensitive colorimetric detection of melamine in milk [47–49]. However, the optimized performance analysis of the colorimetric assay of melamine in milk samples and in aqueous solutions with aptamer-functionalized AuNPs has not been systematically investigated. In this work, we prospected the application of aptamer-functionalized AuNPs in the detection of melamine in milk samples in comparison with the detection of melamine in aqueous solutions. It aims to prove that coordination chemistry is one of the main strategies in colorimetric detection of specific targets based on the AuNPs. Compared with the previous works, our study focuses on detection of lower concentration of melamine. Moreover, the kinetic analysis of this colorimetric assay of melamine using aptamer-functionalized AuNPs was validated. And this strategy involving the coordination chemistry is furthermore equally applicable for complex sample matrixes (such as food) since the presence of additional salts, DNA, proteins and small molecules would significantly affect aggregation of AuNPs.

## Materials and methods

### Reagents

100 μM aptamers (5'-TTTTTTTTTTTTTTTTTTTTTTTTTTTTTTTT-3') were custom synthesized by Sangon Biotechnology Co., Ltd. (Shanghai, China). Sodium carbonate and sodium chloride (NaCl) were purchased from Sinopharm Chemical Reagent Co., Ltd. (Shanghai, China). Ammeline, glucose, fructose, and lactose were purchased from Tianjin Chemical Co., Ltd. (Tianjin, China). Melamine was obtained from Sigma-Aldrich (USA). The liquid milk was purchased from the University supermarket at Henan Agricultural University and this milk is produced in China Mengniu Dairy Company Limited. All solvents and reagents were of analytical grade and were used without further purification. Ultrapure water was used throughout the whole experiment. All glassware used in the experiment were soaked in nitrohydrochloric acid and rinsed thoroughly with water and dried in air before use.

### Pretreatment of milk samples

Firstly, 1.2 mL of 10% trichloroacetic acid and 1.5 mL of chloroform were added into 4 mL of liquid milk. The mixture of liquid milk was carefully poured into the centrifugal tube and agitated for 2 min with a water bath shaker before being placed into an ultrasonic bath for 15 min to reach the reaction equilibrium. The liquid milk suspension was homogenized and then it was further centrifuged at 13000 rpm (revolutions per minute) for 10 min. The supernatant was taken into another centrifugal tube and the 1.0 M Na_2_CO_3_ solution was then added into it to reach the pH of 7.2~7.5. Again, this supernatant was centrifuged at 3000 rpm for 3 min. Finally, the supernatant was stored in the refrigerator for further use.

### Aptamer-functionalized AuNPs via coordination bond

**Fig 1:**
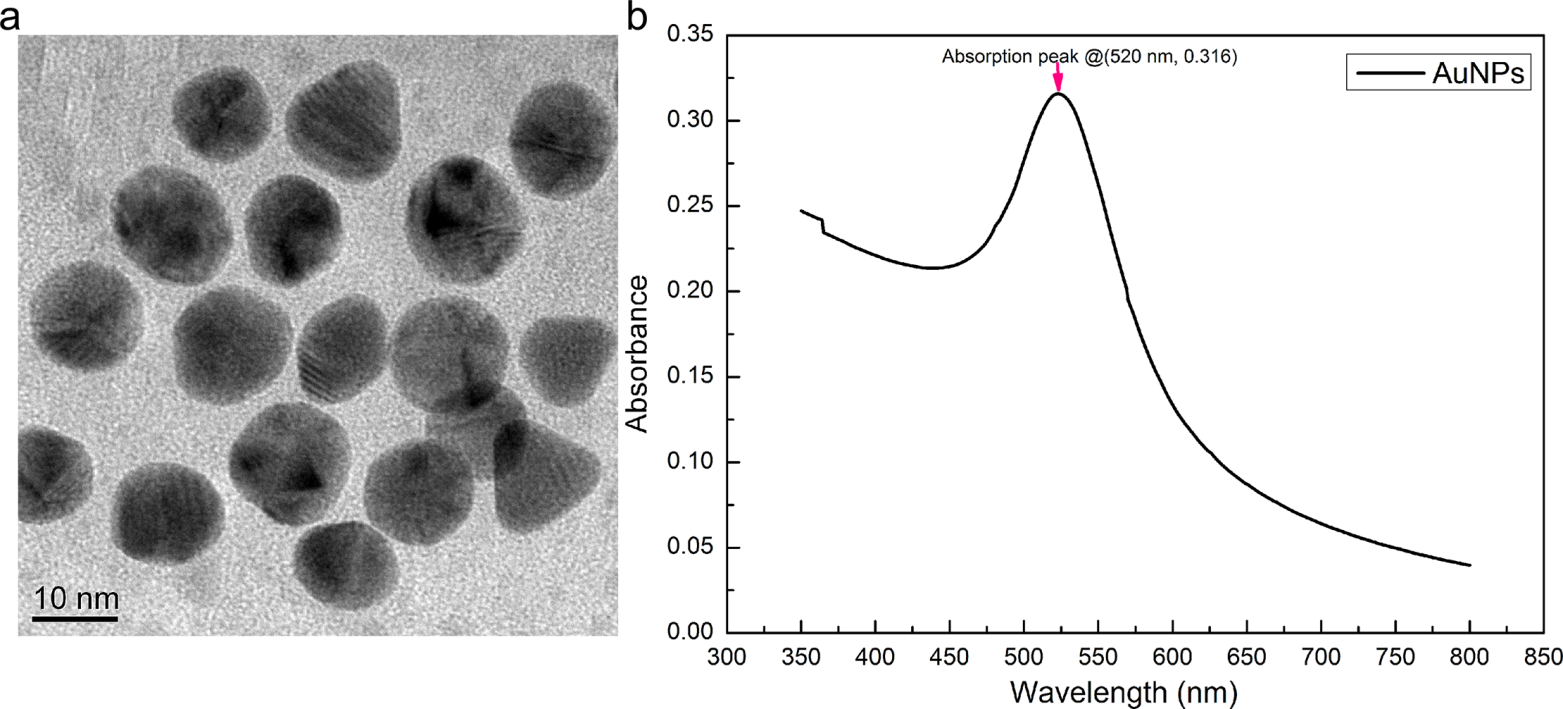
TEM image of the AuNPs solution (a) and UV-Vis absorption spectra of AuNPs solution (b).

We successfully prepared AuNPs with a uniform diameter of 13 nm by following a procedure previously described in the literature [37,46]. The unfunctionalized AuNPs were stable in solution with a wine-red color and has an absorption peak at 520 nm (see Fig 1). In this experiment, AuNPs were coated with the uncoiled single-stranded aptamer (5'-TTTTTTTTTTTTTTTTTTTTTTTTTTTTTTTT-3') by the means of a coordination bond. The aptamer was functionalized with amino (-NH_2_) group on the 5' terminal position. Amino (-NH_2_) groups of aptamers offer a pair of electron that do not participate in bonding. Correspondingly, Au atom in AuNPs provides an empty orbital bonding. Therefore, the aptamers can be absorbed on the surface of AuNPs through the coordination bond. The coordination bond is relatively weak so that the aptamer strands can be desorbed from the surface of AuNPs when target molecules (such as melamine) bind with the aptamer. Here, surface functionalization of AuNPs with aptamers was employed for the colorimetric assay of melamine in milk sample and aqueous solutions to improve the stability of AuNPs at high salt concentrations. When the melamine was added into the aptamer-functionalized AuNPs solution, the melamine molecules were captured by the aptamers through the process of competing association occurring among the AuNPs, aptamers and melamine. In the presence of a certain concentration of NaCl, the aggregation of AuNPs was happened to cause the change of absorbance because the colloid charges were neutralized to weaken the electrostatic force between AuNPs. Aptamer-modified AuNPs were fixed with the transparent 96 well plate cover before the sequential adding of melamine. After 30 min, the aptamer-functionalized AuNPs in each well were washed with a binding buffer, adjusted to have a volume of 200 μL, and the absorption spectra were recorded in the wavelength range of 350~800 nm at25°C using a UV-Vis spectrophotometer (Thermo Fisher Scientific ltd., Finland).

### The principle of colorimetric assay of melamine using aptamer-functionalized AuNPs

**Fig 2:**
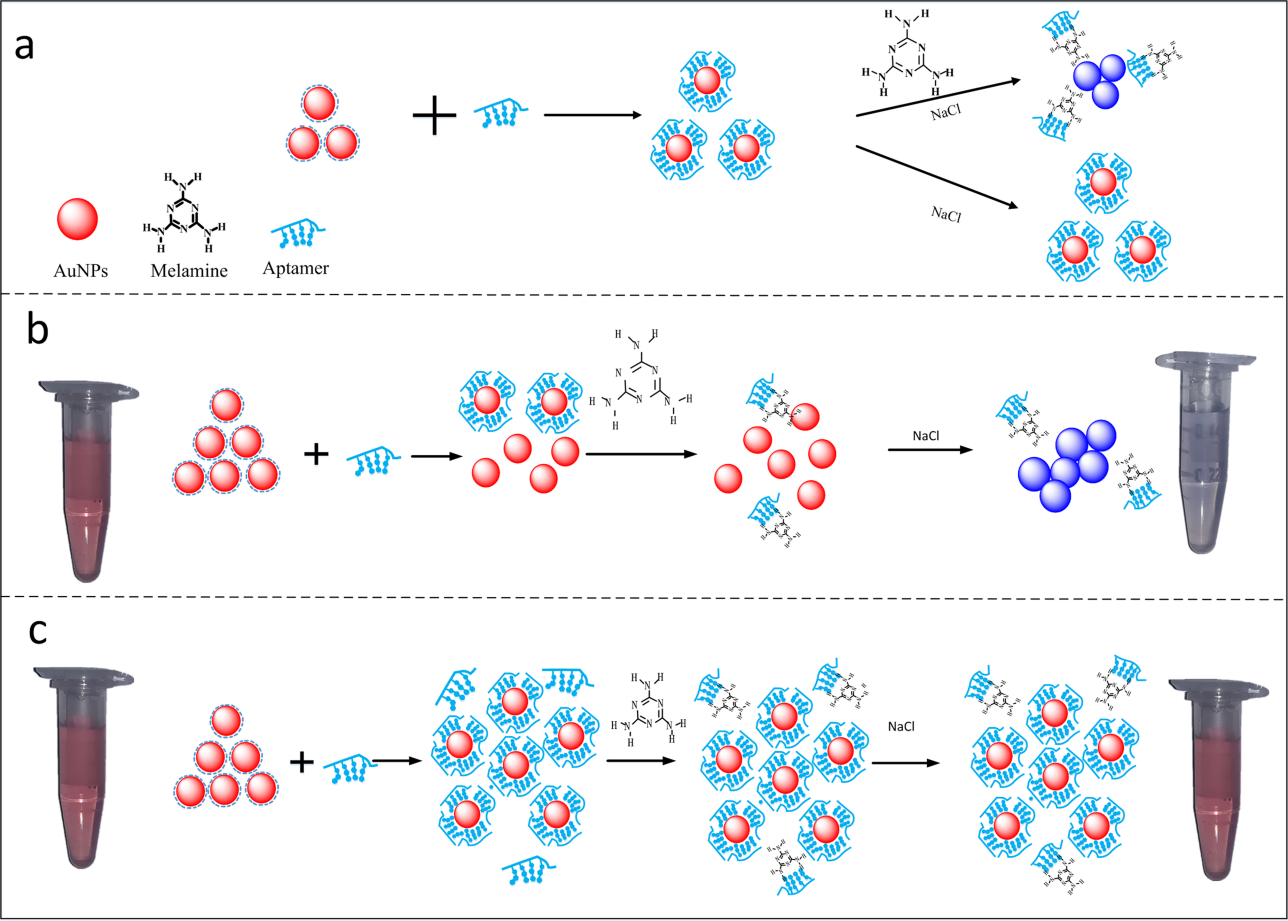
The schematic illustration of the colorimetric assay of melamine using aptamers-functionalized AuNPs.

From the principle of colorimetric assay, generally, the extinction spectrum of the gold sphere can be calculated by solving Maxwell’s equations using the quasi-static approximation [50]:

$$E(\lambda )=\frac{24\pi^{2}N\times a^{3}\times \varepsilon_{d}^{3/2}}{\lambda \times \ln(10)}[\frac{\varepsilon_{i}(\lambda )}{{\left(\varepsilon_{i}(\lambda )+\chi \times \varepsilon_{d}\right)}^{2}+\varepsilon_{i}{\left(\lambda \right)}^{2}}].$$ (1)

where *ε*_*i*_ and *ε*_*d*_ are the dielectric constants of the gold nanoparticle and the external environment, respectively. Since the gold metal is dispersive, its complex dielectric constant is related to the wavelength of incident light *λ*, and can be expressed by *ε*(*λ*) = *ε*_*r*_(*λ*) + *ε*_*i*_(*λ*), where *ε*_*r*_(*λ*) and *ε*_*i*_(*λ*) represent the real and imaginary parts, respectively. *N* is the electron density. χ is the shape factor of the metal nanoparticle for a typical value of 2 for the case of a sphere. The size *a* and shape of AuNPs are closely related to the detection sensitivity. In our experiment, we offer a popular method that relies upon rapid color development in response to aptamer-melamine binding assays using AuNPs. An UV-Vis spectrophotometer (Thermo Fisher Scientific ltd., Finland) provides both qualitative and quantitative information about the functionalized AuNPs solutions. Fig 2 illustrates the principle for detecting melamine in milk samples using aptamer-functionalized AuNPs. Normally, melamine aptamers could be strongly adsorbed on the surface of AuNPs by the electrostatic interaction between the positively charged bases of aptamers and negatively charged AuNPs through the coordination bond, enhancing the stability of AuNPs against the NaCl-induced aggregation in the absence of melamine molecules. However, in the presence of melamine molecules, aptamer-melamine interactions modulate a number of aptamer strands adsorbed on the surface of aptamer-functionalized AuNPs via desorption of the aptamer strands. The aptamers will competitively bind with melamine by the stronger affinity, including the electrostatic effect, hydrogen bond effect, and spatial matching effect, etc. Then in the presence of the salt, salt would neutralize the negative charge of citrate and lead to the AuNPs aggregation. Consequently, the color of AuNPs turns from wine red to blue during the aggregation (see Fig 2A).

In Fig 2A, the aptamer-AuNPs solution is formed by the adsorption of aptamers to AuNPs surface via the coordination bond. In the presence of melamine molecules, the aptamer-melamine interaction results in the desorption of aptamer strands from the AuNPs surface. The increasement of melamine concentration results in a blue shift of the UV-Vis spectra of aggregated AuNPs. The behavior of bonding was shown in the supplementary S1 Fig. In consideration of this colorimetric assay using aptamer-modified AuNPs in practical application, attentions should be paid to the overdose of AuNPs and aptamers (see Fig 2B). When too much amount of AuNPs is used, the certain aptamers can only bind to a small part of AuNPs. In such a case, once the melamine was present in this AuNPs solution, competing association occurred among the AuNPs, aptamers and melamine. The narrow linear range of absorption peak ratio (A650/A520) was obtained at a very low concentration of melamine after the high concentration of NaCl was added into the AuNPs solution. Therefore, it is hard to establish a standard curve for the aptamer-modified AuNPs colorimetric assay of melamine at a normal concentration. On the other hand, if too much amount of aptamers is used in this experiment, the lower limit of detection could not be achieved although the AuNPs were fully covered by aptamers (see Fig 2C). Under normal conditions, the melamine molecules in aptamer-modified AuNPs solution can properly bind to the suspended aptamers due to the strong affinity of aptamer-melamine molecules. In this experiment, 100 μL of 13 nm AuNPs and 40 μL of 1 μM (100 dilutions) aptamers are used, respectively. All measured sample solutions were incubated overnight at room temperature in order to let AuNPs be completely covered with aptamers.

### Standard curves established with known concentrations of melamine in aqueous solution and milk samples

Take 40 μL AuNPs solution with a 96-holeplate specially designed for the UV-Vis spectrophotometer and dilute with both 80 μL ultrapure water and 80 μL milk supernatant, respectively. When 24 μL aptamers were added into the AuNPs solution, the incubation time of aptamers was set to 24 h to make them fully combined. Add 40 μL standard melamine solutions of different concentrations, 0.01 μM, 0.025 μM, 0.05 μM, 0.2 μM, 0.5 μM, 1 μM, and 2 μM, respectively. After mixing with the water bath shaker for 30 min, the 16 μLNaCl solution with the concentration of 500 mM was added to the labeled plate. Each sample solution was vibrated for another 30s before being detected. The absorbance of samples is preserved, and each experimental sample is parallel repeatedly performed three times in parallel. The calibration curve is obtained by using the absorption peak ratio A640/A520 (absorbance at 640 nm and 520 nm) as an ordinate.

## Results and discussion

### The considerations of salt concentrations in AuNPs solutions

**Fig 3:**
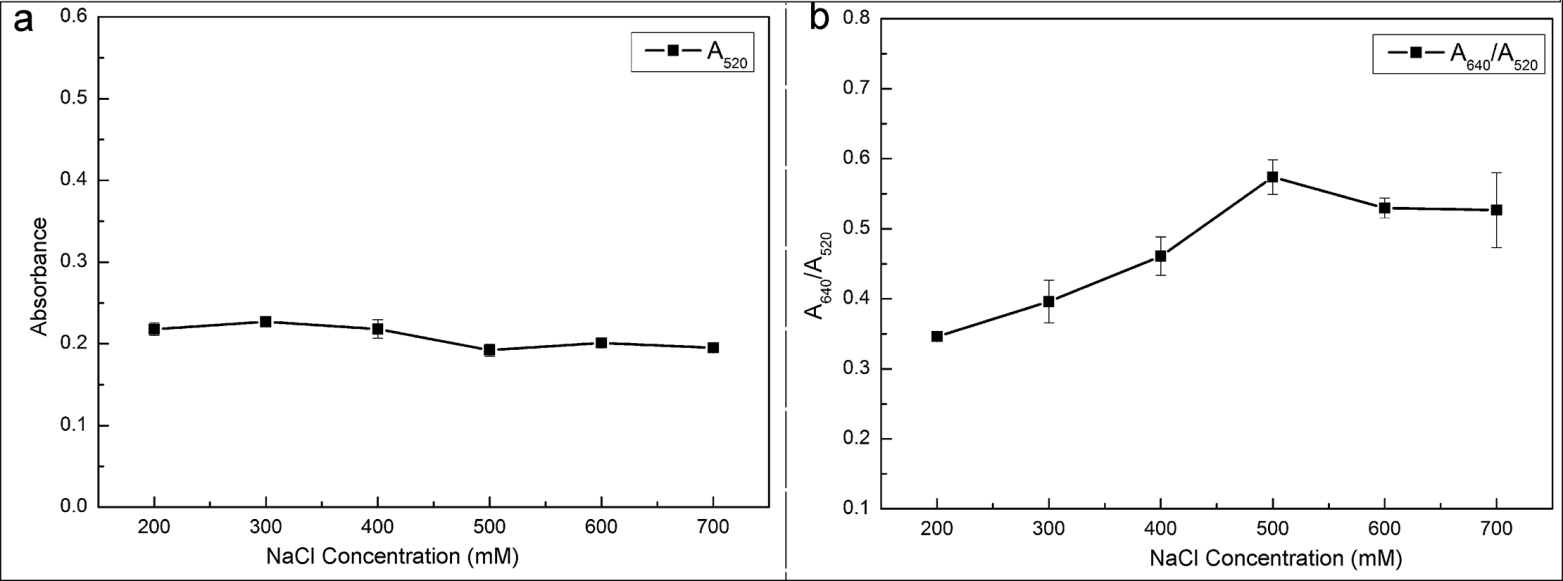
The effect of NaCl concentrations indicated on the absorption spectrum.

The stability of the AuNPs-based colorimetric assay was investigated by adding different concentrations of NaCl into the AuNPs solution in the presence of aptamers (see Fig 3). When the NaCl concentration is too low, the aggregation of AuNPs could not be induced. Correspondingly, the color of the AuNPs solution does not change with the increase of melamine. Contrarily, a high concentration of NaCl can destroy the stability of AuNPs-aptamer. The color of AuNPs solution may already change before the addition of melamine. Therefore, the aggregation of AuNPs is closely related to the concentration of NaCl. At certain concentrations of AuNPs and aptamers, the optimized concentration of NaCl is determined according to the changes of absorption peak ratio (A640/A520) from the absorption spectrum of AuNPs solution. From Fig 3A, the absorbance of the AuNPs solution at 520 nm was not quite different within the wavelength range of 350 ~800 nm, even though the concentration of NaCl was increased. The reason is that the binding process between AuNPs and aptamers was very well after a 24-hour incubation. However, the absorption peak ratio (A640/A520) in the range of 200 mM-500 mM gradually increased with the increase of NaCl concentration initially. Then the absorption peak ratio (A640/A520) decreased slightly and tended to be stable when the concentration of NaCl was more than 500 mM (see Fig 3B). This fact indicated that there was a small amount of unfunctionalized AuNPs left in the solution at the NaCl concentration of 500 mM, even slightly dipped down formed by their weight. Therefore, in this experiment, the 500 mM was chosen as the optimized concentration of NaCl.

### Kinetic analysis of this colorimetric assay of melamine

**Fig 4:**
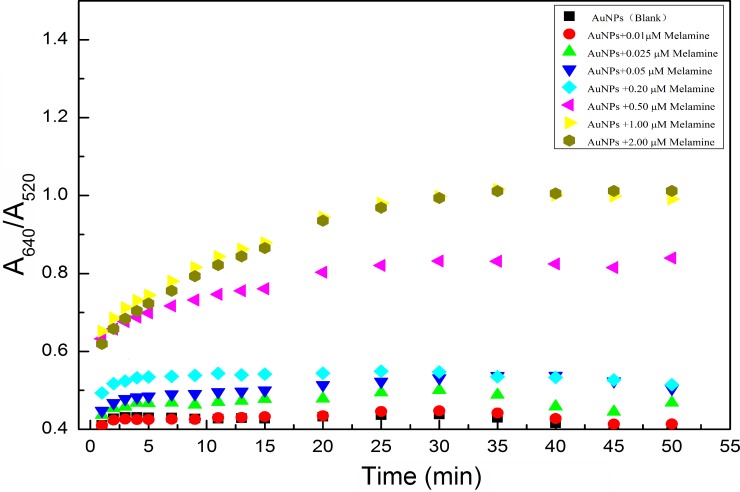
Reaction time with different known concentrations of melamine added intoAuNPs solution.

In order to quantitatively detect melamine using aptamer-functionalized AuNPs as colorimetric probes, a UV-Vis spectroscopy is used to monitor the aggregation of AuNPs induced by melmaine. The absorption ratio (A650/A520) profiles of the AuNPs with different concentrations of melamine are shown in Fig 4.

The spectral curves were taken at different instants of reaction time, 1 min, 2 min, 3 min, 4 min, 5 min, 7 min, 9 min, 11 min, 13 min, 15 min, 20 min, 25 min, 30 min, 35 min, 40 min, 45 min, and 50 min, respectively. By analyzing the kinetics of this colorimetric assay based on aptamer-functionalized AuNPs, the higher the concentration of melamine, the faster the aggregation of AuNPs induced (see Fig 4). At the beginning (less than the reaction time of 5 min), the absorption peak ratio (A640/A520) increased evidently with the increase of reaction time *t* when the concentration of melamine was 0.01 μM, 0.025 μM, 0.05 μM and 0.2 μM, respectively. Nevertheless, the absorption peak ratio (A640/A520) was no longer growing and then rapidly reached to equilibrium when the reaction time *t* was more than 5 min. It indicated that the AuNPs had totally been aggregated due to desorption of aptamers from the surface of AuNPs. On the other hand, the absorption peak ratio (A640/A520) increased with the increase in the reaction time *t* when the concentration of melamine was 0.5 μM, 1 μM and 2 μM, respectively, within the reaction time of 25 min. However, when the reaction time *t* was more than 25 min, the absorption peak ratio (A640/A520) did not increase any more, and the equilibrium is quickly reached. Principally, melamine was completely bonded to the aptamer within a 25 min reaction time. The aptamers were desorbed from the surface of the AuNPs. Then the AuNPs were completely aggregated under the high concentration of NaCl. Fig 4 clearly shows the aggregation of AuNPs at a low concentration of melamine in the beginning of reaction time of 5 min. Therefore, the reaction time of 5 min is enough to measure the low concentration of melamine so as to achieve the purpose of rapid detection of melamine. Actually, in this experiment, the spectral data were recorded within the reaction time of 30 min for accurate analysis due to greater changes of the absorption peak ratio (A640 /A520) during the reaction period.

### Quantitative determination of melamine

**Fig 5:**
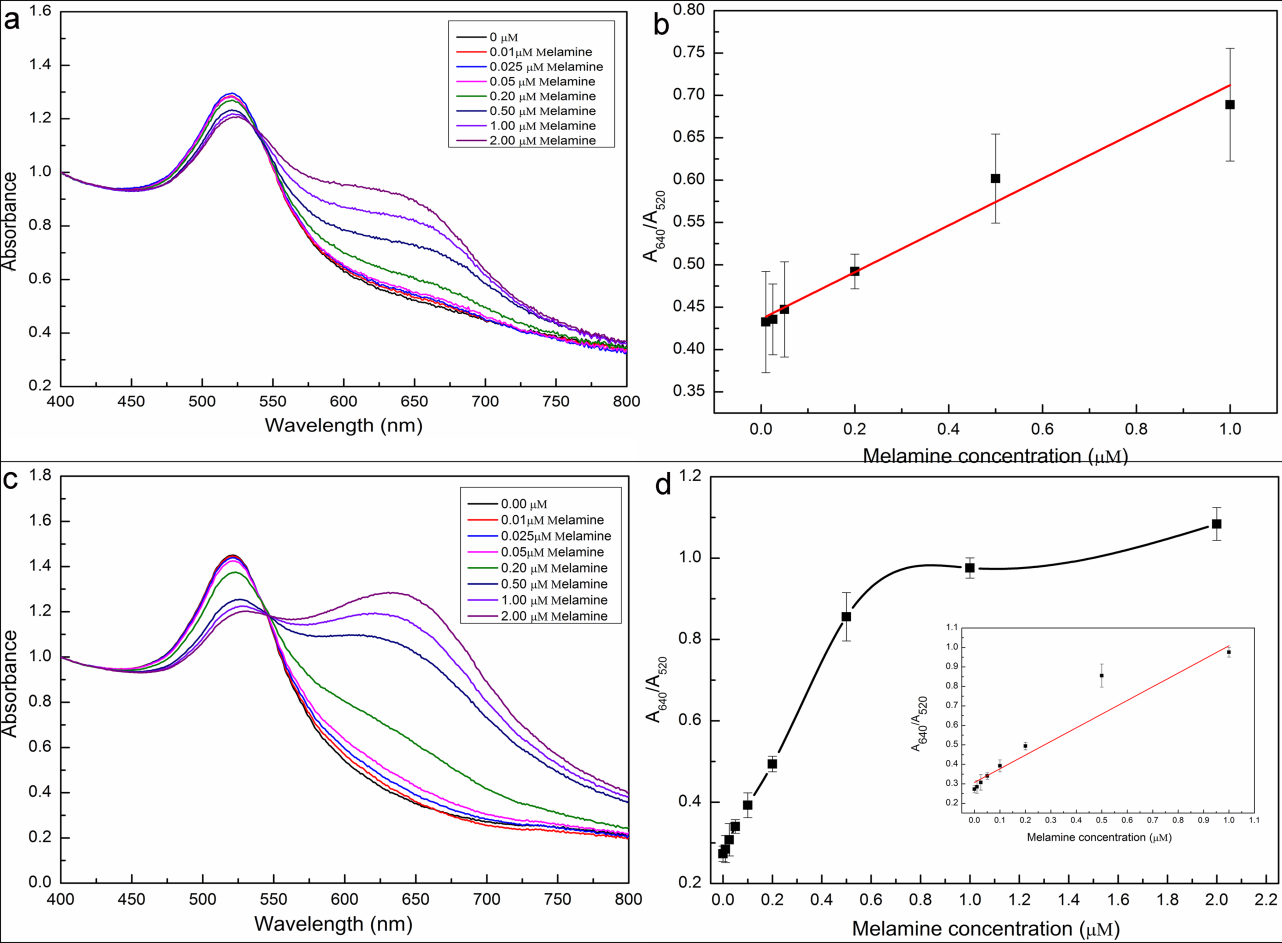
UV-Vis absorption spectra of aptamer-AuNPs solution obtained from different concentrations of melamine samples in aqueous solutions (Fig 5A) and milk samples (Fig 5C), sensitivity of this colorimetric assay system for melamine detection in aqueous solutions (Fig 5B) and in milk solution (Fig 5D), respectively. The concentrations of melamine are 0, 0.1, 0.2, 0.5, 1.0 and 2.0 μM.

To evaluate the reliability of the proposed method, the real melamine-contaminated milk should be tested. In our experiment, the spiked milk samples were taken to evaluate the quantitative results from this colorimetric assay. The different known concentrations of melamine were added into the milk samples and they were prepared by following the preparation procedures described in the previous section of Materials and methods. The substances in the milk samples were well-distributed by mixing with a water bath shaker. After that, the sample solutions with melamine concentrations of 0.025 μM, 0.1 μM and 0.3 μM were obtained. For spiking melamine in milk samples, the desired concentrations were obtained by 5 dilutions of 100 μL accurately prepared sample solutions, which were added into the 400 μL AuNPs solution and mixed evenly. Then 16 μL NaCl at the concentration of 500 mM was added into the AuNPs solution and the mixture is vibrated for 30 s before being measured by the absorption spectroscopy. The normalization of the original spectral data was performed before analyzing (see Fig 5A and Fig 5C). This experiment was repeatedly performed three times. From the UV-Vis absorption spectroscopy, it was observed that the absorbance at 520 nm decreased and a new absorbance peak around 640 nm appeared. The change of UV-Vis absorption spectroscopy is caused by the color change of AuNPs solution from wine red to blue as a result of aggregation with the addition of melamine into AuNPs solution.

Fig 5A and Fig 5C show the spectra obtained from the aptamer-functionalized AuNPs in the aqueous solution and milk samples. The absorption peak ratio (A640/A520) was normalized for analysis. From Fig 5B, when the concentration of melamine was in the range of 0~1μM in aqueous solution, the absorption peak ratio of A640/A520 is linearly correlated with the concentration of melamine. The linear equation is *Y* = 0.272×*C*+0.434 with a linear coefficient (R^2^) of 0.986, where *C* is the concentration of melamine, and *Y* is the value of the absorption peak ratio (A640/A520). According to the 3*σ*/*k* formula (*σ* is the standard deviation of the blank, and *k* is the slope of the linear plot between melamine concentration vs absorption peak ratio of A640/A520), the detection limit (LOD) of this colorimetric assay based on aptamer-functionalized AuNPs was 22 nM of melamine, which was much lower than the LOD (33 nM) obtained by unfunctionalized AuNPs [34]. However, as we can see from the inset of Fig 5D, the absorption peak ratio (A640 /A520) was linearly correlated with the concentration of melamine when the concentration of melamine was less than 0.5 μM. In this case, the corresponding linear equation is expressed as *Y* = 1.157×*C*+0.274 with a linear coefficient (R^2^) of 0.998. However, when the concentration of melamine was larger than 0.5 μM, the absorption peak ratio (A640/A520) increased nonlinearly. This result can be attributed to some compounds in the milk samples that caused the strong light scattering and thus varied the optical path length. The measurements are repeated three times and the results are shown in Table 1. The limit of detections (LODs) of our and other previously reported aptamer-based measurement methods are listed together in Table 2 for the sake of comparison.

**Table 1:** Spiked with different amounts of melamine in milk samples.

Sample	The concentration of melamine (μM)		Recovery rate (%) (n = 3)
	Added melamine	Average measured values	
1	0.025	0.029	117
2	0.1	0.103	103
3	0.3	0.251	84

**Table 2:** A brief list of colorimetric measurement of melamine using aptamer-functionalized AuNPs and unfunctionalized AuNPs.

Method	LOD	Ref.
Colorimetric sensor based on unmodified AuNPs	0.4μg/mL	Li et al. (2010)
Colorimetric aptasensor based on AuNPs	0.5mg/L	Yun et al. (2014)
Aptamers (poly-T10) based on AuNPs	41.7nM	Huang et al. (2011)
Aptamer probe based on AgNPs	3.1mg/L	Liang et al. (2011)
Optical fiber-based localized surface plasmon resonance of AuNPs	33nM	Chang et al. (2017)
Colorimetric measurement of melamine using aptamer-functionalized AuNPs (this work)	14.9nM(in milk sample) 22nM(in aqueous solution)	

### Selectivity experiments

**Fig 6:**
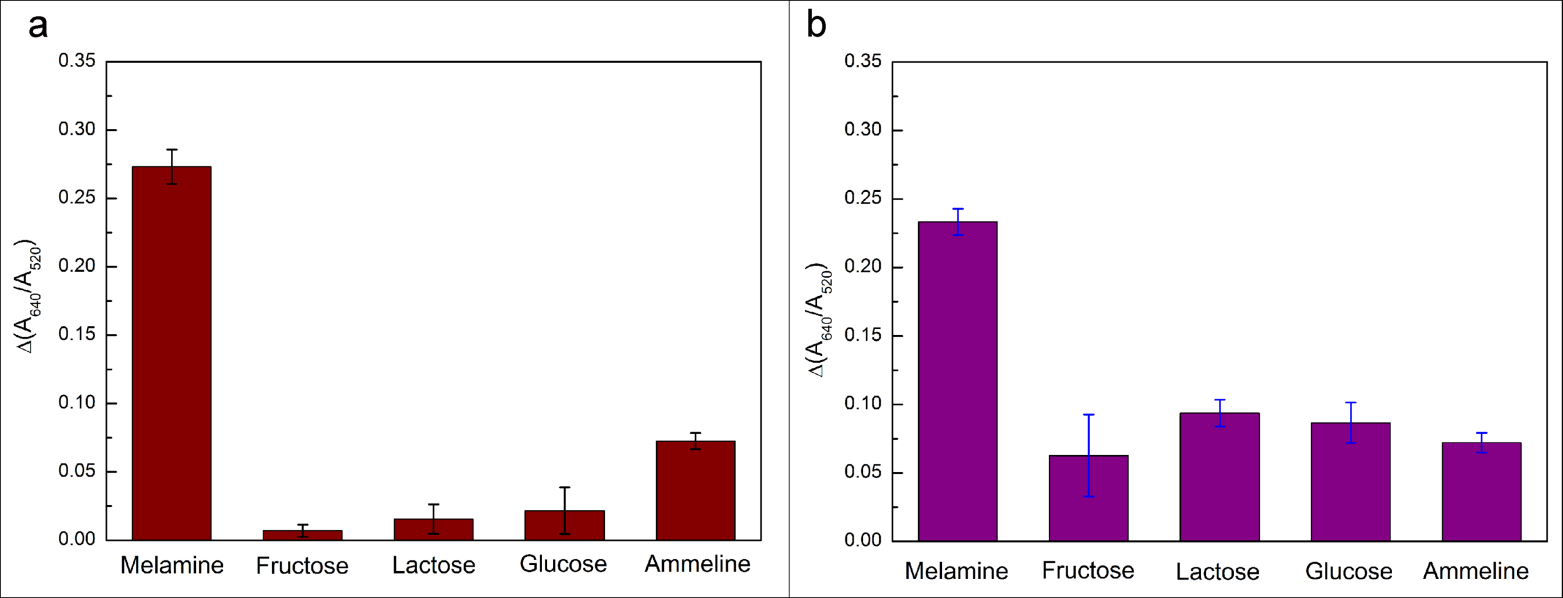
The selectivity of this colorimetric method obtained by adding10 μM of fructose, lactose, glucose and ammeline into the aqueous solution and milk samples, respectively.

In order to determine melamine in milk samples, it is important to consider the interference of other compounds in the milk samples. The selectivity of this method was evaluated in the presence of a much higher concentration 10 μM of fructose, lactose, glucose, and ammeline in aqueous solutions (see Fig 6A) and raw milk samples (see Fig 6B), respectively, when the concentration of standard melamine solution retains 1 μM. The experiment results shown in Fig 6 demonstrated that melamine exhibited the highest absorption peak ratio (A650/A520) and the interference absorption peak ratio of others was roughly an order of magnitude low even if others concentration was 10 times higher than melamine. From this fact, it can be concluded that there is only weak affinity between other compounds with aptamers by few or no hydrogen bond forming. These results proved the efficient selectivity and sensitivity of this colorimetric assay and the possibility of detecting melamine in the raw milk samples.

## Conclusions

In this study, we mainly explore a new way to quantitatively detecting the melamine in milk samples a low concentration below 0.5μM to by an aptamer-functionalized AuNPs colorimetric assay. We proposes the principle of the aptamer-functionalized surface of AuNPs via the desorption of aptamer. The linear responses observed for the absorption peak ratio (A640/A520) are illustrated as functions of melamine concentrations in the spiked milk samples, The results show that the colorimetric assay using aptamer-functionalized AuNPs has a linear detection range of melamine from 0 μM to 0.5 μM in concentration. When compared to the normal measurement system without aptamers, the aptamer-modified AuNPs colorimetric method is more stable and has a better detection limit of 14.9 nM in milk samples. In summary, a new aptamer-modified AuNPs-based colorimetric method was proposed in this work for quantitative detection of melamine from contaminated milk samples. The selectivity of this method was evaluated in the presence of a much higher concentration 10 μM of fructose, lactose, glucose, and ammeline in aqueous solutions and raw milk samples, respectively, when the concentration of standard melamine solution retains 1 μM. The experiment results showed that melamine exhibited the highest absorption peak ratio (A650/A520) and the interference absorption peak ratio of others was roughly an order of magnitude low even if others concentration was 10 times higher than melamine. From the experimental results, this aptamer-modified AuNPs-based colorimetric method is simple, selective, and sensitive for the detection of melamine in milk samples. However, the challenge associated with the detection limit of aptamer-modified AuNPs-based colorimetric method is also existed in the affinity of aptamer to melamine. To further reduce the limit of detection, our group is carrying out some other experiments involving the use of catalytic nanoparticles and aptamers with high affinity. We expect that the method can provide a powerful and universal tool for other molecule-related colorimetric measurement, aptamer, and AuNPs shape studies, not only in studies about target detection but also innumerous.

## Supporting information

S1 Fig (TIF)
