# Supplementary figures and images for: Aptamer-functionalized AuNPs for the high-sensitivity colorimetric detection of melamine in milk samples
Source: PLoS One. 2018 Aug 2;13(8):e0201626. doi: 10.1371/journal.pone.0201626 (PMC6072046; doi:10.1371/journal.pone.0201626)

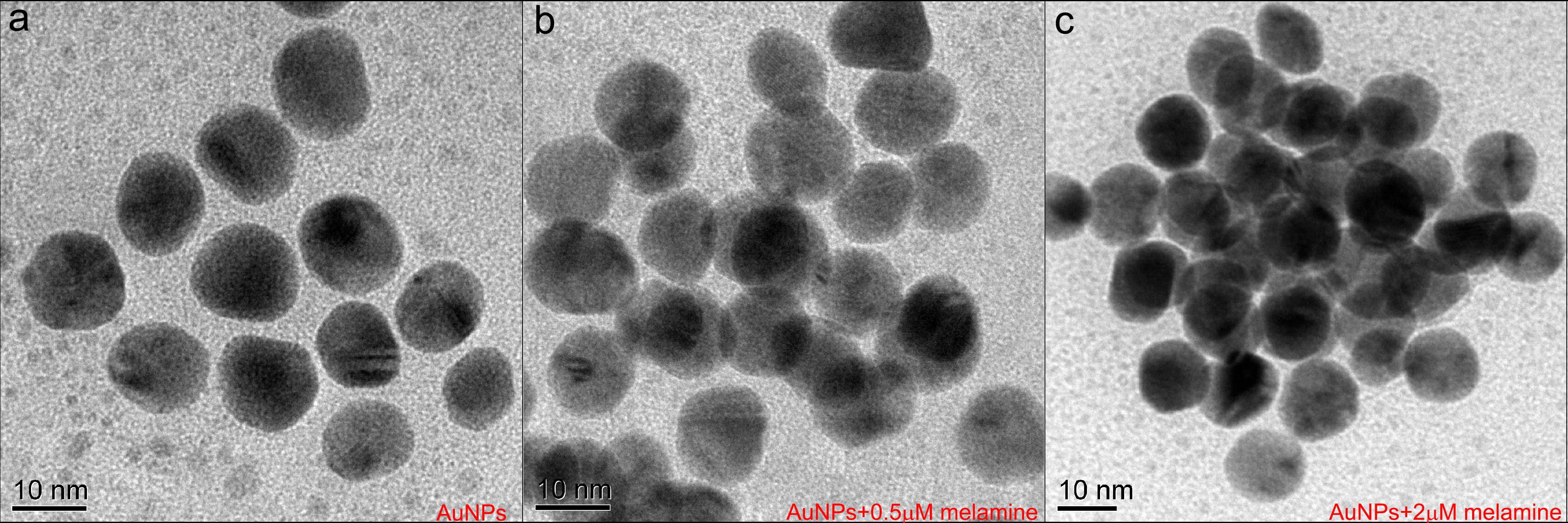

Supplement: S1 Fig — (TIF) [file pone.0201626.s001.tif]
